# Development of a UPLC-TQ/MS Approach for the Determination of Eleven Bioactive Components in Haizao Yuhu Decoction Plus-Minus Haizao and Gancao Drug Combination after Oral Administration in a Rat Model of Hypothyroidism

**DOI:** 10.3390/molecules22010007

**Published:** 2016-12-22

**Authors:** Yingchang Ma, Yang Zhang, Yuanjuan Zhai, Zhenhua Zhu, Ying Pan, Dawei Qian, Shulan Su, Xinsheng Fan, Jinao Duan

**Affiliations:** 1 Jiangsu Collaborative Innovation Center of Chinese Medicinal Resources Industrialization, and National and Local Collaborative Engineering Center of Chinese Medicinal Resources Industrialization and Formulae Innovative Medicine, Nanjing University of Chinese Medicine, Nanjing 210023, China; kittyma128@sohu.com (Y.M.); zy91923@163.com (Y.Z.); zhaiyanjuan1990@126.com (Y.Z.); 04040416@163.com (Z.Z.); ptianxie@126.com (Y.P.); qiandwnj@126.com (D.Q.); sushulan1974@163.com (S.S.); 2 Basic Medical College, Nanjing University of Chinese Medicine, Nanjing 210023, China

**Keywords:** Haizao Yuhu Decoction, hypothyroidism, multiple components, pharmacokinetic differences, UPLC-TQ/MS

## Abstract

Haizao Yuhu Decoction (HYD) has been used for approximately 500 years and is well-known in Traditional Chinese Medicine for its efficacy in the treatment of thyroid-related diseases. In this study, a rapid liquid chromatography-tandem mass spectrometry method was developed for the determination of liquiritin, naringin, hesperidin, peimine, liquiritigenin, glycyrrhizic acid, bergapten, nobiletin, osthole, and glycyrrhetinic acid in rat plasma to investigate the pharmacokinetic profile of different HYD prescriptions in a rat model of hypothyroidism. The differences in pharmacokinetic parameters among the groups were compared by Student’s *t*-test. The pharmacokinetic profile of liquiritin, naringin, hesperidin, peimine, liquiritigenin, glycyrrhizic acid, bergapten, nobiletin, osthole, and glycyrrhetinic acid showed significant differences between Haizao and Gancao anti-drug combination and other herbs in HYD. These results may contribute to the rational clinical use of HYD and reveal the compatibility profile of the Haizao and Gancao anti-drug combination.

## 1. Introduction

Hypothyroidism is the most common pathological hormone deficiency and is more frequent in women than in men. The incidence of hypothyroidism increases with age, especially after mid-life. Populations with a higher risk of developing hypothyroidism include postpartum women [1], individuals with a family history of autoimmune thyroid disorders [2,3] and patients with previous head and neck or thyroid irradiation or surgery, or other autoimmune endocrine conditions. The main treatment for hypothyroidism is supplementation with thyroxine (such as levothyroxine sodium) to achieve restoration of thyroid function [4]. However, due to the narrow toxic-to-therapeutic ratio of thyroid hormone, thyroxine treatment can cause some adverse reactions related to excessive or increased thyroid hormone action, and include symptomatic thyrotoxicosis, subclinical thyrotoxicosis with an increased risk of bone loss [5], and atrial tachyarrhythmias [6]. Compared with thyroxine treatment, Traditional Chinese Medicine (TCM) has shown significant advantages.

Compound TCMs (also known as traditional Chinese formulae) have been used in China for thousands of years. TCM has accumulated more than 100,000 formulae over the past 2000 years [7]. Haizao Yuhu Decoction (HYD) is a formula which has been used for approximately 500 years and is well-known in TCM for its efficiency in treating thyroid-related diseases. HYD was first reported by Chen Shigong in a famous surgical monograph named *Waike Zhengzong* during the Ming Dynasty period. The decoction consists of 10 crude herbs, including *Glycyrrhiza uralensis* Fisch (Gancao), *Sargassum pallidum* (Turn.) C.Ag. (Haizao), *Ecklonia kurome* Okam (Kunbu), *Pinellia ternata* (Thunb.) Breit. (Banxia), *Fritillaria thunbergii* Miq. (Zhebeimu), *Forsythia suspensa* (Thunb.) Vahl (Lianqiao), *Ligusticum chuanxiong* Hort. (Chuanxiong), *Angelica sinensis* (Oliv.) Diels. (Danggui), *Angelica pubescens* Maxim.f. *biserrata* Shan et Yuan (Duhuo) and *Citrus reticulata* Blanco (Chenpi and Qingpi). Modern research has shown that HYD exhibits significant protective effects by reversing serum T_3_, T_4_, TgAb and TPOAb levels, histopathological changes and TRAIL protein expression in the thyroid in rat models of experimental autoimmune thyroiditis (EAT) [8]. HYD with different compatibility can inhibit thyroid enlargement induced by propylthiouracil (PTU), to some extent, and the mechanism may be related to the activation of gene transcription of thyroid peroxidase (TPO) and thyroid globulin [9].

**Figure 1:**
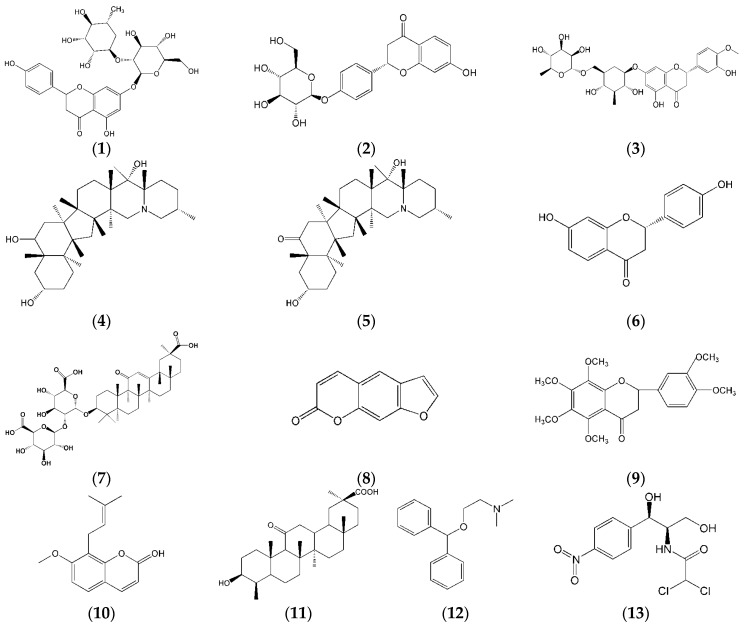
The chemical structures of liquiritin (**1**); naringin (**2**); hesperidin (**3**); peimine (**4**); peiminine (**5**); liquiritigenin (**6**); glycyrrhizic acid (**7**); bergapten (**8**); nobiletin (**9**); osthole (**10**); glycyrrhetinic acid (**11**); diphenhydramine (IS for positive ionization mode) (**12**); and chloromycetin (IS for negative ionization mode) (**13**).

However, the clinical application of HYD is limited due to the Haizao and Gancao drug combination which is considered to be an unfavorable combination according to the “Eighteen antagonisms” (also known as Shibafan in Chinese) which are controversially prohibited combinations in TCM, based on a history of thousands of years of experience. Pharmacokinetic studies of many formulae have reported concerns regarding the drug combinations used in orally administered traditional Chinese recipes [10,11] in terms of their curative effect and the rationality of these drug combinations used in the formulae. In the present study, we aim to develop an effective method of enhancing the level of the Haizao-Gancao drug combination by comparing pharmacokinetic studies of different prescriptions of HYD containing eleven compounds, including liquiritin (**1**), naringin (**2**), hesperidin (**3**), peimine (**4**), peiminine (**5**), liquiritigenin (**6**), glycyrrhizic acid (**7**), bergapten (**8**), nobiletin (**9**), osthole (**10**), and glycyrrhetinic acid (**11**) (Figure 1). Information about the 11 compounds is shown in Table 1.

In this study, a rapid liquid chromatography-tandem mass spectrometry (LC-MS/MS) method was developed for the determination of glycyrrhizinic acid, liquiritin, liquiritigenin, glycyrrhetic acid, hesperidin, naringin, nobiletin, peimine, peiminine, osthole, and bergapten after oral administration of HYD plus-minus Haizao and Gancao drug combination. In addition, pharmacokinetic profile differences in the different prescriptions of HYD in rats were investigated in order to determine the compatibility of the Haizao and Gancao drug combination and other herbs in HYD.

## 2. Results and Discussion

### 2.1. Sample Preparation

Plasma samples (200 μL) and IS solution (50 μL, 1215 ng/mL for diphenhydramine, 476 ng/mL for chloromycetin) were added to an Eppendorf tube, and this mixture was extracted with methanol (550 μL) by shaking on a vortex-mixer for 2 min, and centrifuged for 10 min at 13,000 rpm. The contents were evaporated to dryness in a rotary evaporator at 37 °C. The residue was reconstituted in methanol (100 μL) and centrifuged (13,000 rpm for 10 min). The supernatant was transferred to an autosample vial and an aliquot of 5 μL was injected onto the UPLC-MS/MS system for analysis.

### 2.2. Method Validation

#### 2.2.1. Specificity

**Figure 2:**
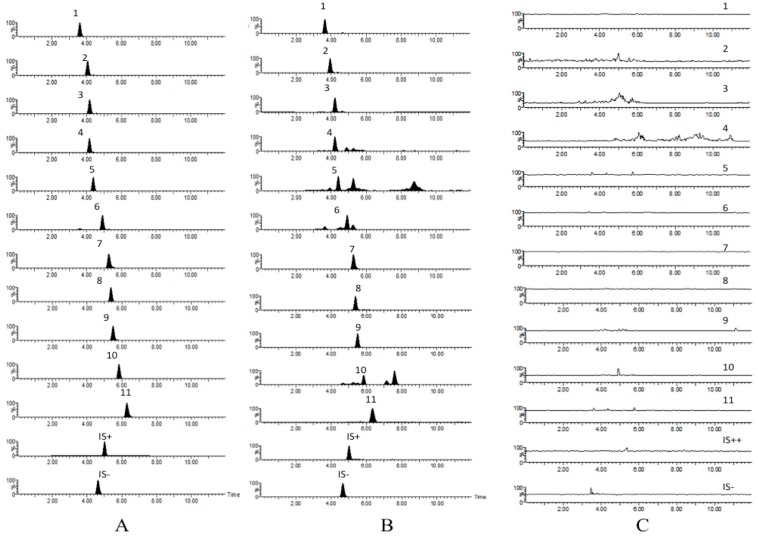
Active extraction MRM chromatograms of compounds **1**–**11**, chloromycetin (IS−) and diphenhydramine (IS+): (**A**) blank plasma spiked with the **11** analytes and IS; (**B**) 15 min sample of plasma after a single oral dose of HYD; (**C**) blank plasma.

All the analytes and internal standards were detected on multiple reaction monitoring (MRM) spectrograms without any endogenous interference (Figure 2).

#### 2.2.2. Linearity and Lower Limits of Quantification (LLOQ)

The calibration curves of the eleven compounds exhibited good linearity with correlation coefficients (R^2^) ranging from 0.9913 to 0.9998. The LLOQs were suitable for quantitative detection of analytes in the pharmacokinetic studies. Linear ranges, LLOQs, LLODs and correlation coefficients are shown in Table 2.

#### 2.2.3. Precision and Accuracy

The results of the intra- and inter-day precision and accuracy of all the analytes in LLOQ and quality control (QC) samples are summarized in Table 3.

The intra- and inter-day precisions ranged from 5.74% to 12.03% and from 4.85% to 11.15%, respectively. The accuracy derived from QC samples was between 89.01% and 104.36% for each QC level of the eleven analytes. The results demonstrated that the precision and accuracy values were within the acceptable range.

#### 2.2.4. Extraction Recovery and Matrix Effect

The mean recoveries of all analytes at different concentrations are shown in Table 4. The extraction recoveries of three level QC samples were more than 65.08%. The extraction recovery of IS was 79.32%–102.77%. The matrix effect of blank plasma in all the analytes was found to be within the acceptable range; all values were more than 66.75% (Table 4). The matrix effect of IS was 98.59%–103.84%. Thus, the plasma matrix effect was demonstrated to be negligible in the assay.

#### 2.2.5. Stability

Stability of the eleven analytes during sample storage and processing procedures was evaluated by analysis of QC samples. The results are shown in Table 5. The findings indicated that these analytes in rat plasma were stable after storage for one month at −80 °C, 24 h in the auto-sampler (4 °C) and three freeze-thaw cycles with accuracy in the range of 68.93%–103.09%.

### 2.3. Pharmacokinetics Study

**Figure 3:**
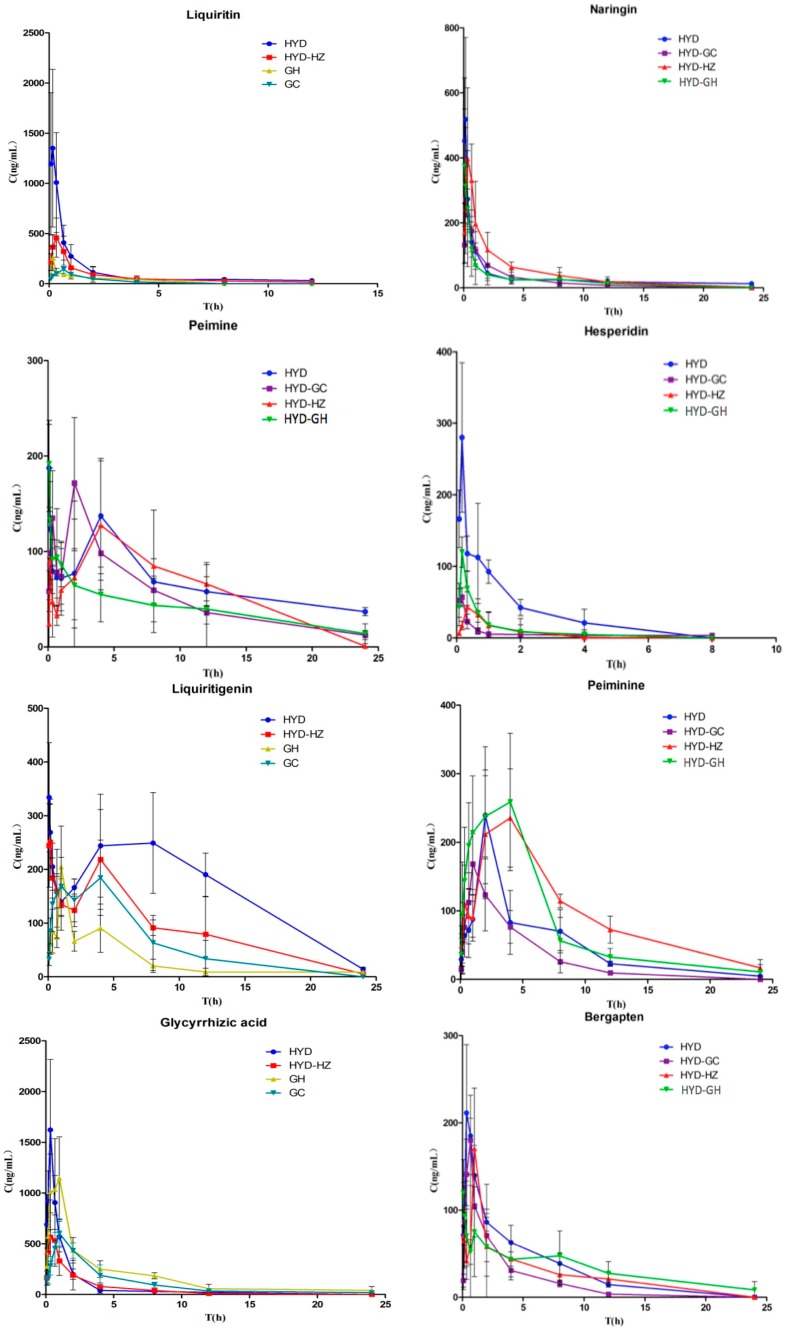

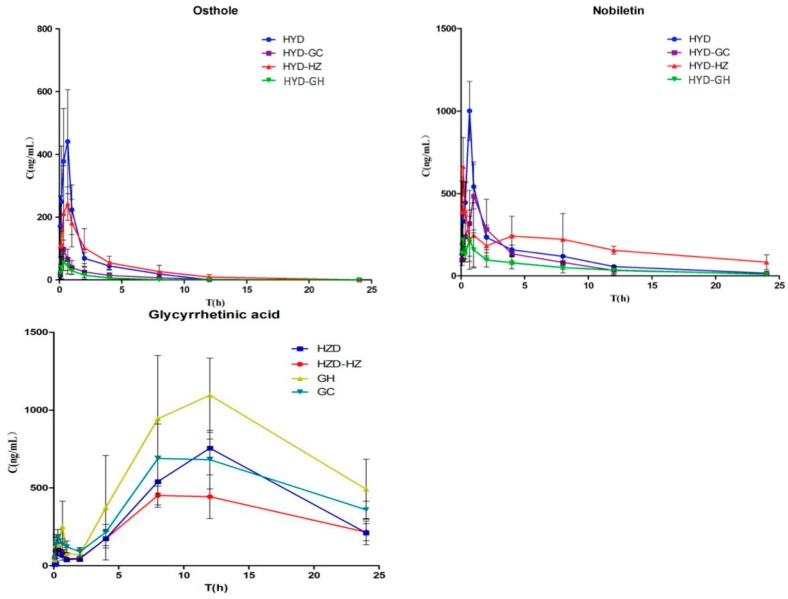
Mean plasma concentration–time curves of eleven compounds in the rat model of hypothyroidism (*n* = 6).

The developed and validated method was used to evaluate the pharmacokinetics of the eleven compounds after oral administration of different fractions in normal rats (Figure 3). The assay was proved to be sensitive enough for the determination of these analytes in rat plasma. The pharmacokinetic parameters including half-life (T_1/2_), maximum plasma concentration (C_max_), time to reach the maximum concentration (T_max_), and area under concentration–time curve (AUC_0~t_) were calculated by a non-compartment model and are shown in Table 6.

#### 2.3.1. Comparison of the Pharmacokinetic Profile of Group GC and GH

The T_max_ values of liquiritin in group GH significantly decreased compared with those in group GC, while the AUC_0~t_ increased as shown in Table 7 (*p* < 0.01). These results suggest that the herb Haizao could have a marked influence on the pharmacokinetic profile of liquiritin in the herb Gancao, it may advance the time of peak value and enhance the bioavailability of liquiritin.

Following co-administration of Gancao and Haizao, the C_max_ and AUC_0~t_ of glycyrrhizinic acid were greater compared with single administration of Gancao, which showed that Haizao increased the absorption of glycyrrhizinic acid. The decrease in AUC value indicated that the herb Haizao could lead to poorer absorption of liquiritigenin in the herb Gancao. Although the pharmacokinetic profile of glycyrrhetinic acid in group GH showed no obvious difference in comparison with that in group GC, the C_max_ of glycyrrhetinic acid increased significantly.

#### 2.3.2. Comparison of the Pharmacokinetic Profile of the GC and HYD-HZ Groups

As shown in Table 8, following HYD-HZ administration in model rats, T_max_, C_max_ and AUC_0~t_ of liquiritin increased significantly, especially T_max_ and C_max_ (*p* < 0.01). Although the T_1/2_ of glycyrrhizinic acid showed no obvious difference in comparison with that in the GC group, its half-life showed a decreasing trend. These findings suggest that the other herbs in HYD may lead to better absorption and greater bioavailability, and delay the peak concentration of liquiritin. The pharmacokinetic profiles of the other three bioactive components in Gancao showed no significant differences.

#### 2.3.3. Comparison of the Pharmacokinetic Profile of the HYD and HYD-GH Groups

As shown in Table 9, the T_1/2_ of naringin, hesperidin, peimine and osthole in the HYD group changed significantly compared with the HYD-GH group. Gancao and Haizao drug combination prolonged elimination of the components in Chenpi, Qingpi, Beimu and Duhuo. In addition, an obvious decrease in the T_max_ value of bergapten was detected. The herb Gancao and Haizao had a marked impact on the absorption of nobiletin and osthole. The C_max_ of nobiletin and osthole as well as the AUC_0~t_ of osthole were greatly reduced in group HYD-GH compared with group HYD.

### 2.4. Double Peak Phenomena

The plasma concentration-time curves of some bioactive ingredients showed double peaks or shoulders which were also reported in previous studies after oral administration of extract [12,13]. Oral administration is the predominant route of drug administration, and there are many factors affecting the absorption of a drug into the bloodstream, which is a very complex process [14]. The cause of the double peak phenomenon after oral administration is controversial and still unclear.

The reason why the plasma concentration-time curves of flavonoids showed a second peak may be due to bacterial metabolism in the intestine [15]. Glycyrrhizinic acid can be metabolized into glycyrrhetinic acid, which is mainly absorbed in the large intestine [16]. This may be the main cause of the second peak seen in the plasma concentration-time curves of glycyrrhetinic acid. However, these hypotheses require further investigation.

### 2.5. Influence of Haizao and Gancao Drug Combination and Other Herbs in HYD on the Pharmacokinetic Profile of the Eleven Bioactive Components

The drug combinations in traditional Chinese recipes can significantly influence the blood concentration and the pharmacokinetic parameters of the individual components after oral administration. In this study, the pharmacokinetic parameter data obtained for liquiritin, naringin, hesperidin, peimine, peiminine, liquiritigenin, glycyrrhizic acid, bergapten, nobiletin, osthole, and glycyrrhetinic acid in the different groups showed some significant differences.

The results indicated that the herb Haizao advanced the time to peak value, enhanced the bioavailability of liquiritin and increased the absorption of glycyrrhizinic acid and glycyrrhetinic acid in Gancao. In addition, the other herbs in HYD also led to greater bioavailability of liquiritin. The effects of Haizao and Gancao drug combination on the pharmacokinetic profile of other herbs in HYD were mainly on T_1/2_, C_max_ and AUC_0~t_. The C_max_ of nobiletin and osthole as well as the AUC_0~t_ of osthole were greatly increased under the influence of Haizao and Gancao.

The cytochrome P450 enzymes (CYPs) belong to a super family of hemeproteins, involved in the metabolism of exogenous and endogenous chemicals [17]. Drug metabolism mainly depends on isozymes of the cytochrome P450 enzyme system, such as CYP1A2 (13%), CYP2C (20%), CYP2D (2%), CYP2E1 (7%), and CYP3A (29%) [18,19,20]. The drug interactions associated with induction or inhibition of CYP enzymes have been proved to be among the most important factors in causing side effects in clinics. Some published papers indicated that glycyrrhetic acid in the herb Gancao inhibited CYP2C19, CYP2C9 and CYP3A4 enzyme activities with inhibitory potencies up to 50% [21]. P-glycoprotein (P-gp) is an energy-dependent membrane protein encoded by multidrug resistance 1 which was first found in tumor cells [22]. Drug concentration can be reduced by P-gp, which is known as an ATP-dependent drug efflux pump [23,24]. Haizao and Gancao drug combination are also thought to be an inhibitor of P-gp. However, Haizao alone may not be an inhibitor of P-gp function in the intestinal membrane, but some of the components of herb Haizao may strengthen membrane permeability to increase the bilateral transport of other components [25]. This may explain why the Gancao and Haizao drug combination prolonged the elimination and enhanced the absorption of the bioactive components in Chenpi, Qingpi, Beimu and Duhuo.

### 2.6. Study limitations

This study has some limitations. The main ingredients in the herb Haizao are meroterpenoids, phlorotannins and polysaccharide [26,27,28]. Most of the ingredients are not indicators as they are mainly endogenous or have not been quantitatively tested by LC-MS/MS. Recently, the pharmacokinetic profile of arsenic (As) in Haizao was studied in our laboratory at different doses using HPLC-HG-AFS [29]. In the herb Haizao, as is present in high concentrations and primarily exists in inorganic forms and is known to be a deadly toxic substance. Further study will be carried out to investigate the effects of herb Gancao on the pharmacokinetic profile of the toxic ingredients in the herb Haizao.

## 3. Experimental Section

### 3.1. Materials and Reagents

HYD includes the following crude drugs: (**1**) *Glycyrrhiza uralensis* Fisch (Gancao); (**2**) *Sargassum pallidum* (Turn.) C.Ag. (Haizao); (**3**) *Ecklonia kurome* Okam (Kunbu); (**4**) *Pinellia ternata* (Thunb.) Breit. (Banxia), (**5**) *Fritillaria thunbergii* Miq. (Zhebeimu); (**6**) *Forsythia suspensa* (Thunb.) Vahl (Lianqiao); (**7**) *Ligusticum chuanxiong* Hort. (Chuanxiong); (**8**) *Angelica sinensis* (Oliv.) Diels. (Danggui); (**9**) *Angelica pubescens* Maxim.f. *biserrata* Shan et Yuan (Duhuo); and (**10**) *Citrus reticulata* Blanco (Chenpi and Qingpi). All materials were purchased from the Fengyuan Pharmaceutical Company of Anhui Province (Hefei, China) and authenticated by Jinao Duan. These materials met the qualitative and quantitative stipulations of the 2010 Chinese Pharmacopoeia. Voucher specimens were deposited in the Herbarium of Nanjing University of Chinese Medicine, Nanjing, China. Acetonitrile and formic acid were of HPLC-grade and obtained from Merck (Darmstadt, Germany) and deionized water was purified using an EPED super purification system (Eped, Nanjing, China). The reference compounds liquiritin (111610-201106), naringin (110722-201312), hesperidin (110721-2001316), peimine (110750-201110), peiminine (110751-201110) and osthole (110822-201308) were purchased from the Chinese National Institute of Pharmaceutical and Biological Products (Beijing, China). Glycyrrhizic acid, glycyrrhetinic acid, nobiletin and bergapten were purchased from Nanjing Spring-Autumn Biological Engineering Co., Ltd. (Nanjing, China). Liquiritigenin (131229) was purchased from Shanghai Winherb Medical Technology Co., Ltd. (Shanghai, China). All other reagents were obtained from Sinopharm Chemical Reagent Co., Ltd. (Nanjing, China), unless otherwise stated.

### 3.2. Animals

All experiments were performed with male Wistar rats, weighing 220–250 g, obtained from the Vital River Experimental Animal Co., Ltd. (Beijing, China). They were kept in plastic cages at 22 ± 2 °C with free access to pellet food and water. Animal welfare and experimental procedures were carried out in accordance with the guide for the Care and Use of Laboratory Animals (National Research Council, Washington, DC, USA), and the Committee for the Update of the Guide for the Care and Use of Laboratory Animals (2011) and related ethical regulations of Nanjing University of Chinese Medicine.

### 3.3. Chromatographic Conditions

Chromatographic analysis was performed using a Waters Acquity UPLC system (Waters Corp., Milford, MA, USA), consisting of a binary pump solvent management system, an online degasser, and an auto-sampler. An Acquity UPLC BEH C 18 (100 mm × 2.1 mm, 1.7 μm) column was used for all analyses. The column temperature was maintained at 35 °C. The mobile phase was composed of (A) formic acid aqueous solution (0.1%); and (B) acetonitrile using a gradient elution of 10%–30% B at 0–4 min, 30%–80% B at 5–9 min, 80%–95% B at 5–9 min, 95% B at 9–10 min, 95%–100% B at 10–11.2 min.

### 3.4. Mass Spectrometry Conditions

Mass spectrometry detection was performed using a Xevo Triple Quadrupole MS (Waters Corp.) equipped with an electro-spray ionization source (ESI). The ESI source was set in both positive and negative ionization mode. The parameters in the source were set as follows: capillary voltage 3.0 kV; source temperature 150 °C; desolvation temperature 550 °C; cone gas flow 50 L/h; desolvation gas flow 1000 L/h. Analyte detection was performed using MRM. The cone voltage and collision energy were optimized for each analyte and selected values are shown in Table 10. All data collected in centroid mode were acquired using Masslynx 4.1 software (Waters Corp.) and post-acquisition quantitative analysis was performed using the TargetLynx program (Waters Corp.).

### 3.5. Preparation of HYD and Omitted Ingredients in HYD

Raw materials of *Glycyrrhiza uralensis* Fisch (Gancao) (200 g) were crushed into small pieces and then refluxed with 2 L water for 1 h and with 1.6 L water for 1 h. The filtrates were combined and concentrated below 70 °C to obtain a certain volume at the ratio of 2:1 (*w*/*w*, weight of all constituting herbs and the extract filtrates) under vacuum. The same method was used to prepare the extract of *Sargassum pallidum* (Turn.) C.Ag. (Haizao), *Ecklonia kurome* Okam (Kunbu), *Pinellia ternata* (Thunb.) Breit. (Banxia), *Fritillaria thunbergii* Miq. (Zhebeimu), *Forsythia suspensa* (Thunb.) Vahl (Lianqiao), *Ligusticum chuanxiong* Hort. (Chuanxiong), *Angelica sinensis* (Oliv.) Diels. (Danggui), *Angelica pubescens* Maxim.f. *biserrata* Shan et Yuan (Duhuo) and *Citrus reticulata* Blanco (Chenpi and Qingpi). A 10 mL extract of Gancao, Haizao, Banxia, Zhebeimu, Lianqiao, Chuanxiong, Danggui, Duhuo, Chenpi, Qingpi and a 5 mL extract of Kunbu were mixed together to prepare the HYD extract. HYD minus Gancao (HYD-GC); HYD minus Haizao (HYD-HZ); HYD minus Gancao and Haizao (HYD-GH); the extract of Gancao and Haizao (GH) and the extract of Gancao (GC) were also prepared. The extracts contained 13.87, 11.73, 16.53, 9.87, 13.84, 11.96, 28.14, 16.94, 10.09, 17.41, 4.19 μg/mL of compounds **1**–**11** in HYD; 0, 11.73, 16.53, 9.87, 13.84, 0, 0, 16.94, 10.09, 17.41, 0 μg/mL of compounds **1**–**11** in HYD-GC; 13.87, 11.73, 16.53, 9.87, 13.84, 11.96, 28.14, 16.94, 10.09, 17.41, 4.19 μg/mL of compounds 1–11 in HYD-HZ; 0, 11.73, 16.53, 9.87, 13.84, 0, 0, 16.94, 10.09, 17.41, 0 μg/mL of compounds **1**–**11** in HYD-GH; 13.87, 0, 0, 0, 0, 11.96, 28.14, 0, 0, 0, 4.19 μg/mL of compounds **1**–**11** in GH, and 13.87, 0, 0, 0, 0, 11.96, 28.14, 0, 0, 0, 4.19 μg/mL of compounds **1**–**11** in GC.

### 3.6. Preparation of Calibration Standards and Quality Control Samples

A standard stock solution mixture containing the eleven compounds was prepared in methanol with a final concentration of 28.2 μg/mL for liquiritin (**1**); 22.7 μg/mL for naringin (**2**); 18.7 μg/mL for hesperidin (**3**); 17.7 μg/mL for peimine (**4**); 18.2 μg/mL for peiminine (**5**); 21.1 μg/mL for liquiritigenin (**6**); 22.2 μg/mL for glycyrrhizic acid (**7**); 21.4 μg/mL for bergapten (**8**); 18.1 μg/mL for nobiletin (**9**); 18.5 μg/mL for osthole (**10**); and 24.5 μg/mL for glycyrrhetinic acid (**11**), respectively. The stock solution was serially diluted with methanol to provide working standard solutions of the desired concentrations. The IS stock solutions (24.6 μg/mL for diphenhydramine—IS for positive ionization mode and 23.8 μg/mL for chloromycetin—IS for negative ionization mode) were also prepared using methanol. IS working solutions (1215 ng/mL for diphenhydramine, 476 ng/mL for chloromycetin) were prepared by diluting the stock solution with methanol. Calibration samples were prepared by mixing solutions of standard mixture, IS and methanol with rat blank plasma to obtain final concentrations in the range of 2.82–2820 ng/mL for liquiritin, 2.27–2270 ng/mL for naringin, 1.87–1870 ng/mL for hesperidin, 1.77–1770 ng/mL for peimine, 1.82–1820 ng/mL for peiminine, 2.11–2110 ng/mL for liquiritigenin, 2.22–2220 ng/mL for glycyrrhizic acid, 2.14–2140 ng/mL for bergapten, 1.81–1810 ng/mL for nobiletin, 1.85–1850 ng/mL for osthole, 1.82–1820 ng/mL for glycyrrhetinic acid, and 1215 ng/mL for diphenhydramine (IS for positive ionization mode) and 476 ng/mL for chloromycetin (IS for negative ionization mode) for IS, respectively. All solutions were stored at −20 °C before use. QC samples were also prepared in the same way (2.82, 141, 2820 ng/mL for liquiritin, 2.27, 114, 1135 ng/mL for naringin, 1.87, 94, 935 ng/mL for hesperidin, 1.77, 88.5, 1770 ng/mL for peimine, 1.82, 91, 910 ng/mL for peiminine, 2.11, 105.5, 1055 ng/mL for liquiritigenin, 2.22, 111, 2220 ng/mL for glycyrrhizic acid, 2.14, 107, 1070 ng/mL for bergapten, 1.81, 90.5, 1810 ng/mL for nobiletin, 1.85, 92.5, 925 ng/mL for osthole and 2.45, 122.5, 2450 ng/mL for glycyrrhetinic acid) at low, middle and high concentrations.

### 3.7. Validation Procedures

The specificity of the method was evaluated by preparing and analyzing six different batches of rat plasma to determine potential interferences at the LC peak region for analytes and IS. The rat plasma chromatograms were compared with those obtained with a sample at the concentration of LLOQ. The signal intensity at this concentration was at least five times higher than that of blank plasma.

The linearity of each calibration curve was determined by plotting the peak area ratio (y) of analytes to IS versus the nominal concentration (x) of analytes with weighted (1/x^2^) least square linear regression.

Accuracy and intra- and inter-day precision were estimated by analyzing three QC samples (five samples for each) at low, middle and high concentrations on the same day and on three consecutive validation days, respectively. The precision was evaluated by relative standard deviation (RSD %) and accuracy by (mean measured concentration/spiked concentration) × 100%.

Extraction recovery was assessed by comparing the peak responses of three QC samples (five samples for each) with the responses of analytes from standard solutions spiked in post-extracted blank plasma at equivalent concentrations.

The matrix effect was measured by comparing the peak responses obtained from samples where the extracted matrix was spiked with standard solutions to those obtained from neat standard solutions at equivalent concentrations.

Three QC samples (five samples for each) were tested for pre-treatment, post-treatment, three freeze-thaw cycles and long-term stabilities. Pre-treatment stability was assessed by exposing QC samples to room temperature for 4 h. Post-treatment stability was evaluated by placing QC samples in the auto-sampler at 4 °C for 24 h. For freeze-thaw cycle stability assessment, QC samples were repeatedly frozen and thawed for three cycles from −80 °C to 20 °C. Long-term stability was carried out by placing QC samples at −80 °C for 2 weeks.

### 3.8. Preparation of Rat Model of Hypothyroidism

A considerable increase in thyroid stimulating hormone (TSH) and decrease in triiodothyronine (T_3_) and tetraiodothyronine (T_4_) in serum are considered to be markers of successful hypothyroid rat model preparation [30]. PTU is the classic drug used to replicate hypothyroidism in rat models. It is generally thought that PTU can decrease the level of thyroid hormone in a hypothyroid rat model by inhibiting the activity of TPO [31,32]. Model Wistar rats were administered PTU intragastrically at a dose of 10 mg/kg (QD) for 2 weeks to establish hypothyroidism. Serum from model rats was collected on the last day of PTU administration and the T_3_, T_4_ and TSH levels were determined using test kits. Wistar rats with a considerable increase in thyroid TSH and a decrease in serum T_3_ and T_4_ were considered to have hypothyroidism and were selected for subsequent pharmacokinetic studies. The levels of these hormones are shown in Table 11.

### 3.9. Pharmacokinetic Studies

For the pharmacokinetic studies, model Wistar rats were divided into six groups (*n* = 6 per group). Rats in the model HYD groups were administered HYD at a dose of 12 mL/kg intragastrically. In the model HYD-GC groups, HYD-GC (11 mL/kg) was administered. In the model HYD-HZ groups, HYD-HZ (11 mL/kg) was administered. In the model HYD-GC-HZ groups, HYD-GC-HZ (10 mL/kg) was administered. In the model GH groups, GH (2 mL/kg) was administered. In the model GC groups, GC (1 mL/kg) was administered. Blood samples were collected at specific time points before (0 min) and after oral administration (5, 10, 20, 40, 60, 120, 240, 480, 720, 1440 min). A total of 360 blood samples were collected. All blood samples were immediately centrifuged at 2500 rpm for 10 min to obtain plasma, which was labeled and frozen at −80 °C until analysis. Blank plasma was obtained from the rats without oral administration and was used to investigate the assay development and validation.

### 3.10. Statistical Analysis

To determine the pharmacokinetic parameters of compounds **1**–**11** in different groups, concentration–time data were analyzed by DAS 3.2 software (Mathematical Pharmacology Professional Committee of China, Shanghai, China, 2011). Data were expressed as the mean and standard deviation (S.D.) with triplicate measurements. The identification of significant differences between different groups was carried out using the Student’s *t*-test. A *p* value < 0.05 was considered statistically significant.

## 4. Conclusions

In this study, a rapid, selective and specific LC-MS/MS method for the simultaneous analysis of eleven components in rat plasma using a 12.0 min simple chromatographic run was developed for the first time. The herb Haizao advanced the time to peak value, enhanced the bioavailability of liquiritin and increased the absorption of glycyrrhizinic acid and glycyrrhetinic acid in Gancao. In addition, the other herbs in HYD led to greater bioavailability of liquiritin. The effects of Haizao and Gancao drug combination on the pharmacokinetic profile of other herbs in HYD were mainly related to T_1/2_, C_max_ and AUC_0~t_. The C_max_ of nobiletin and osthole as well as the AUC_0~t_ of osthole were greatly increased under the influence of Haizao and Gancao. These findings may contribute to the rational clinical use of HYD and also determine the compatibility of the Haizao and Gancao drug combination.

## Figures and Tables

**Table 1:** Information about the eleven analyzed compounds.

Name	Biological Effects	Plants Derived
Liquiritin	Antidepressant effects, neuroprotective effects, and effects on the cardiac system	*Glycyrrhiza uralensis* Fisch.
Naringin	Hypolipidemic, sedative, antioxidation, antifungal, antiatherosclerosis, antispasmodic, analgesic, regulation of blood sugar	*Citrus reticulata* Blanco
Hesperidin	Anti-inflammatory effect, prevent osteoporosis, anti-allergic effect, antibacterial and antiviral action, anti-ulcer effect, anti-cancer effect, immunoregulation effect, anti-inflammatory effect	*Citrus reticulata* Blanco
Peimine	On bacterial or leukemic cell multidrug resistance reversal effect, anti-tumor effect	*Fritillaria thunbergii* Miq.
Peiminine	Anti-tumor effect	*Fritillaria thunbergii* Miq.
Liquiritigenin	Anti-virus effect, anti-ulcer and antispasmodic effect	*Glycyrrhiza uralensis* Fisch.
Glycyrrhizic acid	Anti-inflammatory, anti-lipid peroxidation, regulation of immune, stable lysosomal control of liver damage, anti-allergic effect, detoxification effect of glucocorticoid	*Glycyrrhiza uralensis* Fisch.
Bergapten	Antitussive, asthma, expectorant effect	*Forsythia suspensa* (Thunb.) Vahl.
Nobiletin	Anti-inflammatory effect, anti-oxidation, anti-tumor effect, anti-cardiovascular disease, improvement of metabolic disorders, treatment of neurodegenerative diseases	*Citrus reticulata* Blanco
Osthole	Antihypertensive, anti-bacterial effect, anti-viral effect, anti-inflammatory and anti-tumor effects	*Angelica pubescens* Maxim.f. *biserrata* Shan et Yuan
Glycyrrhetinic acid	Anti-oxidation, anti-Gram-negative bacteria and various types of cancer	*Glycyrrhiza uralensis* Fisch.

**Table 2:** The regression equations, linear ranges, LLOQs, and LODs of the eleven compounds.

Compound No.	Linea Regression Equation	R^2^	Range (ng/mL)	LOD (ng/mL)	LOQ (ng/mL)
**1**	*y* = 646.06*x* + 30.033	0.9988	2.82–2820	1.41	2.82
**2**	*y* = 2616*x* + 13.524	0.9939	2.27–2270	1.14	2.27
**3**	*y* = 9615.7*x* + 5.3263	0.9989	9.35–1870	1.87	9.35
**4**	*y* = 230.29*x* + 25.525	0.9949	1.77–1770	0.89	1.77
**5**	*y* = 351.88*x* + 7.7625	0.9976	1.82–1820	0.91	1.82
**6**	*y* = 1189.5*x* + 4.0037	0.9998	2.11–2110	0.89	1.77
**7**	*y* = 18272*x* + 23.182	0.9963	2.22–2220	1.11	2.22
**8**	*y* = 239.92*x* + 4.0037	0.9976	2.14–2140	1.07	2.14
**9**	*y* = 29.156*x* + 15.998	0.9913	1.81–1810	0.91	1.81
**10**	*y* = 170.11*x* + 10.18	0.9933	1.85–1850	0.93	1.85
**11**	*y* = 642.85*x* − 62.082	0.9917	11.1–2220	2.22	11.1

**Table 3:** Precision and accuracy of determination of the four compounds in rat plasma.

Compound No.	Concentration (ng/mL)	Intra-Day		Inter-Day	
		Accuracy (%)	Precision (RSD, %)	Accuracy (%)	Precision (RSD, %)
**1**	2.82	87.00	7.91	79.09	8.19
	1.41 × 10^2^	89.93	6.31	85.58	7.12
	2.82 × 10^3^	87.05	7.99	87.48	7.93
**2**	2.27	73.65	10.31	80.15	7.85
	1.14 × 10^2^	85.73	9.50	88.88	9.13
	1.14 × 10^3^	90.34	9.51	89.87	6.73
**3**	1.87	78.16	6.93	77.93	10.19
	9.35 × 10	81.13	6.85	78.82	10.08
	9.35 × 10^2^	82.46	9.99	80.03	10.03
**4**	1.77	83.56	7.65	90.03	7.11
	8.85 × 10	90.14	5.74	99.13	7.01
	1.77 × 10^3^	93.37	10.03	99.00	8.16
**5**	1.82	92.03	12.03	90.73	9.93
	9.10 × 10	99.75	10.23	93.64	8.15
	9.10 × 10^2^	97.09	10.10	93.94	4.85
**6**	2.11	95.19	10.12	98.53	7.32
	1.06 × 10^2^	97.77	10.30	99.70	8.94
	1.06 × 10^3^	95.30	9.15	100.12	8.94
**7**	2.22	98.56	9.15	95.53	7.95
	1.11 × 10^2^	98.61	8.99	99.01	8.18
	2.22 × 10^3^	98.03	8.96	99.08	8.29
**8**	2.14	90.00	9.81	89.73	7.51
	1.07 × 10^2^	93.05	9.13	98.10	8.29
	1.07 × 10^3^	91.14	10.05	95.34	8.09
**9**	1.81	85.37	10.31	87.03	8.24
	9.05 × 10	90.18	10.95	91.93	8.28
	1.81 × 10^3^	90.29	9.14	91.00	10.73
**10**	1.85	75.39	10.53	79.99	10.03
	9.25 × 10	85.91	9.65	86.54	11.15
	9.25 × 10^2^	98.73	6.13	88.76	9.21
**11**	1.82	81.05	6.17	86.08	5.88
	9.10 × 10	88.89	10.33	91.13	9.03
	1.82 × 10^3^	93.07	7.95	90.35	9.25

**Table 4:** Recoveries and matrix effects of the eleven compounds in rat plasma.

Compound No.	Concentration (ng/mL)	Recovery		Matrix Effect	
		Accuracy (%)	Precision (RSD, %)	Accuracy (%)	Precision (RSD, %)
**1**	2.82	65.08	15.97	66.75	7.21
	1.41 × 10^2^	76.68	13.03	89.40	9.01
	2.82 × 10^3^	77.94	8.93	87.77	7.89
**2**	2.27	75.37	10.35	74.29	8.18
	1.14 ×10^2^	81.09	6.13	89.13	7.66
	1.14 ×10^3^	88.93	9.22	91.01	7.94
**3**	1.87	70.53	10.13	88.18	6.51
	9.35 × 10	81.45	9.56	90.15	9.18
	9.35 ×10^2^	90.06	9.44	90.93	9.10
**4**	1.77	69.97	7.07	89.37	13.95
	8.85 × 10	70.93	7.93	88.16	11.03
	1.77 × 10^3^	79.83	6.54	95.43	9.75
**5**	1.82	71.95	11.98	89.73	10.57
	9.10 × 10	85.54	11.99	92.05	10.66
	9.10 × 10^2^	89.78	10.29	95.96	8.59
**6**	2.11	73.18	7.53	81.55	11.64
	1.06 × 10^2^	89.54	8.17	88.94	10.75
	1.06 × 10^3^	99.17	2.91	93.75	10.16
**7**	2.22	72.34	8.93	74.58	8.15
	1.11 × 10^2^	77.95	8.79	80.13	8.34
	2.22 × 10^3^	86.76	8.10	92.22	8.03
**8**	2.14	81.59	11.57	78.93	7.58
	1.07 × 10^2^	87.63	12.63	91.54	6.85
	1.07 × 10^3^	103.05	11.59	99.78	11.06
**9**	1.81	79.07	5.79	78.65	8.31
	9.05 × 10	81.59	8.73	82.25	7.98
	1.81 × 10^3^	101.15	5.12	89.94	8.82
**10**	1.85	69.34	8.57	73.54	11.13
	9.25 × 10	77.08	7.70	84.75	7.91
	9.25 × 10^2^	86.45	5.94	88.63	8.54
**11**	1.82	83.11	8.91	84.39	15.34
	9.10 × 10	102.53	11.35	89.15	12.68
	1.82 × 10^3^	100.78	7.65	103.76	5.94

**Table 5:** Stability of the eleven compounds in rat plasma.

Compound No.	Concentration (ng/mL)	Freeze-Thaw Cycles		At −80 °C for a Month		Auto-Sampler for 24 h	
		Accuracy (%)	Precision (RSD, %)	Accuracy (%)	Precision (RSD, %)	Accuracy (%)	Precision (RSD, %)
**1**	2.82	95.34	8.48	78.53	9.15	91.54	9.43
	1.41 × 10^2^	89.93	7.63	81.91	8.18	93.18	9.67
	2.82 × 10^3^	95.99	9.33	99.31`	7.91	93.22	9.13
**2**	2.27	87.95	10.34	91.39	9.99	91.98	10.38
	1.14 × 10^2^	89.34	12.09	93.27	10.28	94.45	11.00
	1.14 × 10^3^	91.16	10.94	99.78	6.59	101.03	7.38
**3**	1.87	81.94	10.75	92.63	6.63	103.05	6.97
	9.35 × 10	93.87	11.50	92.66	8.69	97.09	8.24
	9.35 × 10^2^	99.63	6.13	99.18	9.17	98.48	8.19
**4**	1.77	95.36	7.04	89.15	8.01	87.53	9.17
	8.85 × 10	99.37	15.13	87.63	11.37	89.79	12.58
	1.77 × 10^3^	99.25	11.59	95.44	10.13	89.99	8.88
**5**	1.82	90.35	8.19	75.93	11.30	75.55	9.48
	9.10 × 10	97.59	11.57	92.26	6.59	87.63	5.33
	9.10 × 10^2^	97.71	8.15	103.15	9.27	87.61	9.23
**6**	2.11	87.55	11.68	81.23	9.69	80.18	9.17
	1.06 × 10^2^	86.19	8.87	83.34	11.34	89.91	8.62
	1.06 × 10^3^	78.13	8.93	81.96	5.98	93.13	6.18
**7**	2.22	68.93	7.52	79.95	11.97	81.39	9.74
	1.11 × 10^2^	81.54	8.91	78.18	8.13	83.57	8.11
	2.22 × 10^3^	79.96	7.11	103.09	8.09	87.79	7.18
**8**	2.14	71.63	11.88	85.34	13.09	94.53	8.81
	1.07 × 10^2^	96.15	7.89	89.92	7.08	96.97	6.53
	1.07 × 10^3^	96.03	7.14	91.00	5.90	99.90	5.44
**9**	1.81	90.23	12.08	87.34	7.12	91.81	13.13
	9.05 × 10	91.52	10.93	88.16	7.09	95.58	10.95
	1.81 × 10^3^	94.47	13.67	95.84	7.24	76.17	8.91
**10**	1.85	90.57	9.14	88.85	8.96	76.17	8.91
	9.25 × 10	90.78	11.00	89.93	6.93	88.00	13.59
	9.25 × 10^2^	92.79	8.35	92.38	6.65	89.64	11.65
**11**	1.82	89.87	8.79	85.13	15.53	83.29	11.94
	9.10 × 10	91.34	7.69	90.03	9.18	92.23	10.87
	1.82 × 10^3^	99.69	8.12	99.36	8.36	101.43	9.92

**Table 6:** Pharmacokinetic parameters of compounds **1**–**11** after oral administration of different decoctions in normal rats (*n* = 6).

Compound No.	Group	C_max_/ng·mL^−1^	T_max_/h	T_1/2_/h	AUC_0~t_/ng·h·mL^−1^
**1**	HYD	1595.00 ± 595.18	0.19 ± 0.11	9.23 ± 5.85	1762.25 ± 286.64
	HYD-HZ	493.56 ± 164.61 **	0.31 ± 0.07 **	3.66 ± 3.30	987.22 ± 566.92 *
	GH	254.11 ± 121.96	0.17 ± 0 **	1.34 ± 1.48	2509.26 ± 814.35 **
	GC	147.34 ± 37.69	0.67 ± 0	0.99 ± 0.34	229.19 ± 118.70
**2**	HYD	612.10 ± 200.05	0.17 ± 0.09	11.33 ± 4.86	911.65 ± 197.89
	HYD-GC	271.49 ± 75.41	0.19 ± 0.07	1.12 ± 0.28	561.47 ± 278.27
	HYD-HZ	462.81 ± 193.77	0.50 ± 0.18	2.59 ± 2.02	1060.95 ± 243.51
	HYD-GH	423.79 ± 188.45	0.15 ± 0.10	1.23 ± 0.15 ^##^	565.30 ± 351.95
**3**	HYD	302.93 ± 95.12	0.24 ± 0.21	1.15 ± 0.34	359.29 ± 120.84
	HYD-GC	67.96 ± 9.34	0.13 ± 0.05	1.51 ± 0	85.93 ± 41.99
	HYD-HZ	46.81 ± 21.32	0.44 ± 0.17	0.21 ± 0.12	52.82 ± 56.56
	HYD-GH	119.82 ± 21.24 ^##^	0.17 ± 0	0.52 ± 0.50 ^#^	100.09 ± 44.85 ^##^
**4**	HYD	119.89 ± 68.18	4.38 ± 4.76	30.56 ± 13.26	3795.89 ± 2093.85
	HYD-GC	196.55 ± 54.12	1.42 ± 0.91 *	5.40 ± 1.47	1280.46 ± 287.94
	HYD-HZ	358.38 ± 104.88	0.17 ± 0	7.64 ± 2.44	825.21 ± 81.47
	HYD-GH	122.63 ± 0.15	0.17 ± 0.15	4.52 ± 0.56 ^##^	501.22 ± 414.00 ^#^
**5**	HYD	197.09 ± 115.14	3.17 ± 2.56	3.44 ± 2.84	1170.96 ± 569.74
	HYD-GC	169.17 ± 45.26	1.11 ± 0.46	1.75 ± 0.99	768.95 ± 246.38
	HYD-HZ	274.30 ± 76.06	2.08 ± 1.68	5.82 ± 2.46	2292.23 ± 244.80
	HYD-GH	302.24 ± 62.36	3.11 ± 1.44	4.70 ± 1.96	1043.15 ± 549.78
**6**	HYD	357.96 ± 91.94	2.75 ± 4.07	4.05 ± 0.77	39.27.33 ± 554.59
	HYD-HZ	275.66 ± 66.54	0.79 ± 1.58	3.94 ± 0.59	2146.24 ± 629.49
	GH	211.11 ± 70.27	1.50 ± 1.23	4.12 ± 3.73	681.38 ± 771.03 *
	GC	220.40 ± 47.43	2.50 ± 1.73	2.41 ± 1.57	1000.77 ± 945.93
**7**	HYD	1373.60 ± 711.55	0.292 ± 0.10	1.58 ± 1.01	1689.27 ± 638.54
	HYD-HZ	587.45 ± 261.23	0.67 ± 0.42	1.84 ± 1.82	587.45 ± 261.23
	GH	1345.54 ± 321.92 **	0.56 ± 0.40	9.94 ± 8.89	3597.01 ± 1839.69 *
	GC	607.13 ± 137.38	1.25 ± 0.50	4.47 ± 2.63	1762.95 ± 1426.16
**8**	HYD	226.76 ± 57.80	0.33 ± 0.21	1.56 ± 1.02	819.43 ± 25.72
	HYD-GC	183.32 ± 46.99	0.61 ± 0.14	1.17 ± 0.10	469.69 ± 129.75
	HYD-HZ	191.83 ± 54.52	0.81 ± 0.34	1.92 ± 1.75	649.05 ± 132.42
	HYD-GH	125.28 ± 35.61	0.11 ± 0.04 ^##^	9.60 ± 10.61	785.69 ± 369.20 ^##^
**9**	HYD	1000.95 ± 178.16	0.67 ± 0	6.03 ± 1.95	2865.70 ± 285.38
	HYD-GC	501.42 ± 182.99	0.89 ± 0.27	4.53 ± 1.89	2005.59 ± 1023.79
	HYD-HZ	321.98 ± 88.25	0.19 ± 0.07	16.18 ± 7.06	2293.06 ± 55.53
	HYD-GH	311.16 ± 126.03 ^##^	0.46 ± 0.37	3.26 ± 3.53	1182.52 ± 539.49 ^##^
**10**	HYD	457.96 ± 184.11	0.61 ± 0.14	1.25 ± 0.03	748.45 ± 224.96
	HYD-GC	101.03 ± 31.86	0.39 ± 0.14	1.54 ± 0.51	204.75 ± 94.67
	HYD-HZ	294.65 ± 116.62	0.61 ± 0.25	1.45 ± 1.11	294.65 ± 116.62
	HYD-GH	69.22 ± 25.57 ^##^	0.53 ± 0.31	0.68 ± 0.49 ^#^	98.46 ± 40.96 ^##^
**11**	HYD	504.27 ± 100.76	9.33 ± 2.07	3.01 ± 2.35	9987.32 ± 1663.21
	HYD-HZ	756.10 ± 58.12	12.00 ± 0	1.99 ± 0.03	12,692.39 ± 1489.22
	GH	1247.75 ± 210.12 *	12.67 ± 5.89	1.93 ± 0.04	22,825.00 ± 10,422.15
	GC	776.14 ± 184.15	10.00 ± 2.31	4.66 ± 5.33	10,451.95 ± 8308.34

Data are expressed as mean ± S.D. (*n* = 6); * Difference compared with the corresponding GC group, *p* < 0.05; ** Difference compared with the corresponding GC group, *p* < 0.01; ^#^ Difference compared with the corresponding HYD group, *p* < 0.05; ^##^ Difference compared with the corresponding HYD group, *p* < 0.01.

**Table 7:** Comparison of the Pharmacokinetic Profile of Group GC and GH.

Compound No.	Group	C_max_/ng·mL^−1^	T_max_/h	T_1/2_/h	AUC_0~t_/ng·h·mL^−1^
**1**	GH	254.11 ± 121.96	0.17 ± 0 **	1.34 ± 1.48	2509.26 ± 814.35 **
	GC	147.34 ± 37.69	0.67 ± 0	0.99 ± 0.34	229.19 ± 118.70
**6**	GH	211.11 ± 70.27	1.50 ± 1.23	4.12 ± 3.73	681.38 ± 771.03 *
	GC	220.40 ± 47.43	2.50 ± 1.73	2.41 ± 1.57	1000.77 ± 945.93
**3**	GH	1345.54 ± 321.92 **	0.56 ± 0.40	9.94 ± 8.89	3597.01 ± 1839.69 *
	GC	607.13 ± 137.38	1.25 ± 0.50	4.47 ± 2.63	1762.95 ± 1426.16
**4**	GH	1247.75 ± 210.12 *	12.67 ± 5.89	1.93 ± 0.04	22,825.00 ± 10,422.15
	GC	776.14 ± 184.15	10.00 ± 2.31	4.66 ± 5.33	10,451.95 ± 8308.34

* Difference compared with the corresponding GC group, *p* < 0.05; ** Difference compared with the corresponding GC group, *p* < 0.01

**Table 8:** Comparison of the Pharmacokinetic Profile of the GC and HYD-HZ Groups.

Compound No.	Group	C_max_/ng·mL^−1^	T_max_/h	T_1/2_/h	AUC_0~t_/ng·h·mL^−1^
**1**	HYD-HZ	493.56 ± 164.61 **	0.31 ± 0.07 **	3.66 ± 3.30	987.22 ± 566.92 *
	GC	147.34 ± 37.69	0.67 ± 0	0.99 ± 0.34	229.19 ± 118.70
**6**	HYD-HZ	275.66 ± 66.54	0.79 ± 1.58	3.94 ± 0.59	2146.24 ± 629.49
	GC	220.40 ± 47.43	2.50 ± 1.73	2.41 ± 1.57	1000.77 ± 945.93
**3**	HYD-HZ	587.45 ± 261.23	0.67 ± 0.42	1.84 ± 1.82	587.45 ± 261.23
	GC	607.13 ± 137.38	1.25 ± 0.50	4.47 ± 2.63	1762.95 ± 1426.16
**4**	HYD-HZ	756.10 ± 58.12	12.00 ± 0	1.99 ± 0.03	12,692.39 ± 1489.22
	GC	776.14 ± 184.15	10.00 ± 2.31	4.66 ± 5.33	10,451.95 ± 8308.34

* Difference compared with the corresponding GC group, *p* < 0.05; ** Difference compared with the corresponding GC group, *p* < 0.01

**Table 9:** Comparison of the Pharmacokinetic Profile of the GC and HYD-HZ Groups.

Compound No.	Group	C_max_/ng·mL^−1^	T_max_/h	T_1/2_/h	AUC_0~t_/ng·h·mL^−1^
**2**	HYD	612.10 ± 200.05	0.17 ± 0.09	11.33 ± 4.86	911.65 ± 197.89
	HYD-GH	423.79 ± 188.45	0.15 ± 0.10	1.23 ± 0.15 ^##^	565.30 ± 351.95
**3**	HYD	302.93 ± 95.12	0.24 ± 0.21	1.15 ± 0.34	359.29 ± 120.84
	HYD-GH	119.82 ± 21.24 ^##^	0.17 ± 0	0.52 ± 0.50 ^#^	100.09 ± 44.85 ^##^
**4**	HYD	119.89 ± 68.18	4.38 ± 4.76	30.56 ± 13.26	3795.89 ± 2093.85
	HYD-GH	122.63 ± 0.15	0.17 ± 0.15	4.52 ± 0.56 ^##^	501.22 ± 414.00 ^#^
**5**	HYD	197.09 ± 115.14	3.17 ± 2.56	3.44 ± 2.84	1170.96 ± 569.74
	HYD-GH	302.24 ± 62.36	3.11 ± 1.44	4.70 ± 1.96	1043.15 ± 549.78
**8**	HYD	226.76 ± 57.80	0.33 ± 0.21	1.56 ± 1.02	819.43 ± 25.72
	HYD-GC-HZ	125.28 ± 35.61	0.11 ± 0.04 ^##^	9.60 ± 10.61	785.69 ± 369.20 ^##^
**9**	HYD	1000.95 ± 178.16	0.67 ± 0	6.03 ± 1.95	2865.70 ± 285.38
	HYD-GH	311.16 ± 126.03 ^##^	0.46 ± 0.37	3.26 ± 3.53	1182.52 ± 539.49 ^##^
**10**	HYD	457.96 ± 184.11	0.61 ± 0.14	1.25 ± 0.03	748.45 ± 224.96
	HYD-GH	69.22 ± 25.57 ^##^	0.53 ± 0.31	0.68 ± 0.49 ^#^	98.46 ± 40.96 ^##^

^#^ Difference compared with the corresponding HYD group, *p* < 0.05; ^##^ Difference compared with the corresponding HYD group, *p* < 0.01.

**Table 10:** Precursor/production pairs and parameters for MRM of compounds **1**–**11** used in this study.

Analyte	Retention Time (min)	Ionization Mode	MRM Transitions (Precursor-Product)	Cone Voltage (V)	Collision Energy (eV)
liquiritin	3.60	ESI−	416.99→255.14	28	20
naringin	4.06	ESI−	579.31→271.12	40	28
hesperidin	4.18	ESI+	611.31→303.18	20	46
peimine	4.18	ESI+	432.43→95.15	18	14
peiminine	4.36	ESI+	430.42→98.02	40	46
liquiritigenin	4.92	ESI−	257.15→119.04	26	22
glycyrrhizic acid	5.29	ESI−	823.35→453.35	18	30
bergapten	5.37	ESI+	216.97→89.55	26	22
nobiletin	5.52	ESI+	403.23→183.01	34	48
osthole	5.83	ESI+	245.18→189.05	18	14
glycyrrhetinic acid	6.27	ESI+	480.99→105.10	40	40

**Table 11:** The T_3_, T_4_ and TSH levels in serum of the model rats in different groups.

Group	T_3_ (ng/mL)	T_4_ (ng/mL)	TSH (μIU/mL)
Blank	0.89 ± 0.33	80.63 ± 9.94	4.11 ± 0.94
HYD	0.67 ± 0.17	60.14 ± 8.59	5.61 ± 0.12
HYD-GC	0.48 ± 0.19	65.29 ± 7.29	5.99 ± 0.44
HYD-HZ	0.44 ± 0.39	66.57 ± 9.38	5.12 ± 0.39
HYD-GH	0.51 ± 0.14	70.58 ± 5.51	4.88 ± 0.51
GH	0.66 ± 0.09	61.20 ± 4.12	5.62 ± 0.21
GC	0.52 ± 0.14	72.91 ± 9.08	5.16 ± 0.19
